# Monitoring and Evaluation of the Corrosion Behavior in Seawater of the Low-Alloy Steels BVDH36 and LRAH36

**DOI:** 10.3390/ijms25126405

**Published:** 2024-06-10

**Authors:** Adrian Mazilu, Lidia Benea, Elena Roxana Axente

**Affiliations:** 1 Competences Centre—Interfaces-Tribocorrosion-Electrochemical Systems (CC-ITES), “Dunărea de Jos” University of Galati, 47 Domneasca Street, RO-800008 Galati, Romania; adrian.mazilu@ugal.ro; 2 Faculty of Medicine and Pharmacy, “Dunărea de Jos” University of Galați, 35 Al. I. Cuza Street, RO-800010 Galati, Romania; elena.axente@ugal.ro

**Keywords:** corrosion, sea environment, electrochemical impedance spectroscopy, polarization resistance, low-alloy steels

## Abstract

The purpose of this study is to evaluate the corrosion resistance in natural seawater (Năvodari area) of two types of low-alloy carbon steels BVDH36 and LRAH36 by electrochemical methods. The electrochemical methods used were the evolution of the free potential (OCP), electrochemical impedance spectroscopy (EIS), polarization resistance (R_p_) and corrosion rate (V_corr_), potentiodynamic polarization (PD), and cyclic voltammetry (CV). The studies were completed by ex situ characterization analyzes of the studied surfaces before and after corrosion such as: optical microscopy, scanning electron microscopy and X-ray diffraction analysis. The results of the study show us that the polarization resistance of the low-alloy carbon steel BVDH36 is higher compared to the polarization resistance of the low-alloy carbon steel LRAH36. It is also observed that with the increase in the immersion time of the samples in natural seawater, the polarization resistance of the BVDH36 alloy increases over time and finally decreases, and for the carbon steel LRAH36 the polarization resistance increases.

## 1. Introduction

In a world where metals and metal alloys are indispensable, corrosion appears as one of the most serious problems of modern society and the resulting losses every year are of the order of trillions of dollars [1]. Regarding the corrosion losses, approximately 90% are related to ferrous materials, used in the construction of road bridges, reinforcements, rolling stock and agricultural equipment, maritime ships, and port facilities [2]. In second and third place in the hierarchy of practical use are aluminum and copper alloys, which are also subject to corrosion [2]. Marine corrosion is a permanent problem, not only for moving or stationary ships, but also for all port installations, desalination installations, and objects that come into prolonged contact with water and the marine atmosphere [3,4,5,6,7,8,9,10]. The corrosivity of seawater depends on many factors such as salinity, dissolved oxygen, temperature, biological factors, and the material subject to corrosion, etc. [8]. Depending on the metallic material subjected to the action of seawater, all these factors can also determine the type of corrosion that can occur [8]. Corrosion in the marine environment can manifest in various forms and takes place through electron transfer reactions [8]. That is why predicting the lifetime of a material used in a certain application is particularly important to avoid accidental and catastrophic failures [3,4,5,6,7,8,9,10]. The deep sea is the direction of oceanic exploration due to the wealth of oil, gas, and minerals. In the last decade, a new challenge is focused on the study of the reliability of deep engineering equipment [11,12,13,14]. There has been interest in using low-alloy steels for deep-sea applications due to their excellent mechanical properties, general corrosion resistance, biocompatibility, and weldability in the marine environment [15,16]. Low-alloy steel usually protects other metallic materials by corroding as a sacrificial anode due to the negative self-corrosion potential [17,18,19]. Wei et al. [20] highlighted the fact that low-alloy steels with a carbon content of less than 0.2% by weight, to which Cu, Cr, Ni, P, Si, and Mn are mainly added as alloying elements up to a total of at most 5% by weight, are inexpensive and widely used in marine engineering such as ship plates, sea crossing bridges, and submarine pipelines [20]. However, the marine environment is the most corrosive natural environment, and the service performance of low-alloy steels in marine environments can be difficult to guarantee [20]. Therefore, there is a need to improve the corrosion resistance of low-alloy steels for marine engineering. According to the literature data, it is known that the corrosion resistance of low-alloy steel is closely related to the alloying elements, but the research conclusions are mainly qualitative, such as the following: the corrosion resistance of low-alloy steel will improve with the increase in the content of Cr [20]. Thus, authors such as Jia et al. [21] provided more information on the mechanical properties of low-alloy steel subjected to uniform and pitting corrosion. This study was conducted because in recent times more and more emphasis has been placed on the use of high-strength low-alloy steel [21,22] in the development of long-lasting tall structures or in other special structures, since they contribute to the reduction in element sizes, especially the thickness of the plates. However, if these low-alloy steel structures were to serve in a harsh environment such as a high humidity and salinity atmosphere, they would be exposed to a severe threat of degradation as a result of the corrosion process. In the case of atmospheric corrosion of structures, pitting corrosion and uniform corrosion are very common. However, pitting corrosion is more harmful because it has random characteristics and the maximum stress always occurs at the location of the maximum corrosion depth [23]. Pitting corrosion would induce the degradation of tensile strength [24,25,26,27,28], compressive strength [25,26,27,29,30], flexural strength [24,26,31,32,33], and shear strength [34] of low-alloy steel plate [35]. Also, the reduction in strength is also mainly related to the corrosion process with an effect on the damage of the minimum cross-section. Thus, pitting corrosion leads to a rough surface and contributes to a severe stress concentration [23,36,37], causing a reduction in deformability. The authors [21] highlighted that the prediction relationship of surface roughness and deformability reduction could be described by a parabolic law [24,27]. The location and dimensions of the pitting affect the tensile characteristic and failure mode of the steel plate under load [29]. In this context, Jia et al. [21] performed several copper acetate accelerated spray corrosion tests for high-strength low-alloy steel plate samples. The authors performed the tensile tests in order to investigate the mechanical properties of the low-alloy steel plate before and after corrosion, aiming at the main parameters of corrosion time and tensile prestressing. The study was carried out to establish the relationship between the degradation effects of the low-alloy steel structures due to the two types of corrosion and the analyzed mechanical properties. The experimental results showed that as the corrosion time increased, the total corrosion loss of the unprestressed samples continued to increase with a gradually decreasing corrosion rate. In addition, during corrosion, the roughness experienced an upward trend, and the lower surface was rougher than the upper surface. Separating the corrosion components, the total corrosion loss was dominated by pitting corrosion, although the proportion of uniform corrosion increased more and more. Regarding long-term corrosion, the authors [21] showed that elastic tensile prestressing can lead to some inhibition of pitting corrosion while contributing to the easy development of uniform corrosion. The results of this study [21] showed that compared to the unprestressed specimens, the tensile mechanical and deformation characteristics of the tensile elastically prestressed specimens were both slightly improved. Considering the effects of the two types of corrosion on the mechanical properties, Jia et al. showed that uniform corrosion mainly affected tensile mechanical characteristics, while pitting corrosion is predominantly attributed to deformation performance degradation [21]. Zhang et al. pointed out that the poor corrosion resistance of steel can affect the stability and safety of the material. Therefore, it is extremely important to explore the corrosion behavior of low-alloy steel for practical applications [38]. According to the literature data, it is known that many factors affect the corrosion resistance of low-alloy steels, such as: alloy content [39,40], Cl concentration [41,42], O_2_ content [43,44], corrosion environment [45,46], heat treatment [47,48], and grain size [49,50]. Zhang et al. [38] investigated the effect of tempering temperature on the corrosion of low-alloy steel. Low-alloy steel is mainly supplied in an annealed condition. Although tempering is an effective method of improving the mechanical properties of steel, its influence on corrosion behavior has been controversial. Therefore, Zhang et al. [38] studied the effects of different tempering temperatures on the corrosion resistance of low-alloy steel in humid atmosphere using an immersion test, a salt spray test, electrochemical measurements, morphological characterization, and phase analysis. The results showed that the defects and highly reactive nature of the high angle grain boundaries of “bare” steels are preferentially corroded. In the initial stage of corrosion, the annealing temperature affects the distribution of small-angle grain boundaries. The annealed specimens corroded slightly due to the low interface energy. In the later stage of corrosion, Zhang et al. [38] observed that the corrosion resistance of the analyzed samples still depends on the matrix due to the limited protection of the rust layer. Thus, the load transfer resistance improves and the corrosion resistance becomes better as the annealing temperature decreases. Chen et al. [51] studied literature data on the corrosion mechanism of low-alloy steel in a tropical coastal atmosphere. The authors highlighted in their study that the atmospheric corrosion process is an electrochemical process that occurs when a thin layer of electrolyte forms on the metal surface [52]. The liquid layer with airborne salt species (e.g., chlorides and carbonates) dissolving in coastal atmospheres can greatly increase the corrosion rate of low-alloy steel, especially in tropical regions characterized by a high temperature and relative humidity [53,54,55]. The role of chlorides in the coastal atmospheric corrosion process is extremely complicated and there are several possible mechanisms of acceleration: (a) hygroscopic chlorides would absorb moisture from the atmosphere and thus favor the formation of electrolytes, lowering the relative humidity threshold for corrosion in the atmosphere [56,57]; (b) some chloride rust phases are soluble and therefore the electrolyte conductivity would have increased considerably, which in turn accelerates the atmospheric corrosion process [51]; (c) Cl^−^ (chloride ions) would favor phase transformations in the deposited corrosion products (rust) and therefore could modify the protective functions [58,59]. Based on the information presented above, the authors [51] pointed out that chlorides influence not only the corrosion rate, but also the constituents of the corrosion products. In general, the main corrosion products of low-alloy steel are lepidocrocite (γ-FeOOH) and goethite (α-FeOOH) under low or no Cl^−^ exposure [60,61]. Akaganeite (β-FeOOH) and maghemite (γ-Fe_2_O_3_)/magnetite (Fe_3_O_4_) could be produced by the hydrolysis of ferrous chloride (FeCl_2_) and oxidation of the low-alloy steel substrate relative to a large amount of chloride deposition [62,63]. Among the aforementioned rust phases, the compact and denser α-FeOOH is of critical importance in reducing atmospheric corrosion [59]. Compared to other corrosion products, β-FeOOH is electrochemically active and tends to form thick layers in corrosion products favoring the formation of cracks in these corrosion products as well as localized attack on the surface of low-alloy steel [64,65,66,67,68]. Based on the results of the study [51], it was indicated that a high rate of chloride deposition would reduce the protective capacity of the rust layer due to an increase in the content of harmful akaganeite (β-FeOOH) and maghemite (γ-Fe_2_O_3_) in the corrosion products. Also, the existence of the corrosion products β-FeOOH and γ-Fe_2_O_3_ induced the appearance of pits due to severe pitting corrosion on the surface of low-alloy steel. Quantitatively, strong positive correlations were obtained between the chloride deposition rate, the corrosion rate, and the protective ability index of the rust layer by means of the Pearson correlation coefficient. Thus, the rate of chloride deposition could be directly related to the protection of the rust layer as well as the corrosion severity of low-alloy steel in the tropical coastal atmosphere [51]. The objective of this work is to study the corrosion behavior in natural seawater (Black Sea) of two types of steel (BVDH36 and LRAH36) used in the Galati and Constanţa shipyards for the construction of different parts of maritime ships. In recent years, studies have been reported in the specialized literature that deal more with the influence of technological and mechanical parameters on the corrosion resistance of different types of steel immersed in solutions simulating the marine environment [69,70,71,72,73,74,75,76,77,78,79,80]. But it can be seen that there is a lack of studies regarding the comparison between two types of materials immersed in natural seawater that are focused on obtaining a clearer vision of the corrosion resistance of these types of materials. The paper presents the study of the corrosion behavior of the two steels by electrochemical methods such as the free potential, electrochemical impedance spectroscopy, potentiodynamic polarization, and cyclic voltammetry.

## 2. Results and Discussion

### 2.1. The Evolution of the Free Potential

**Figure 1:**
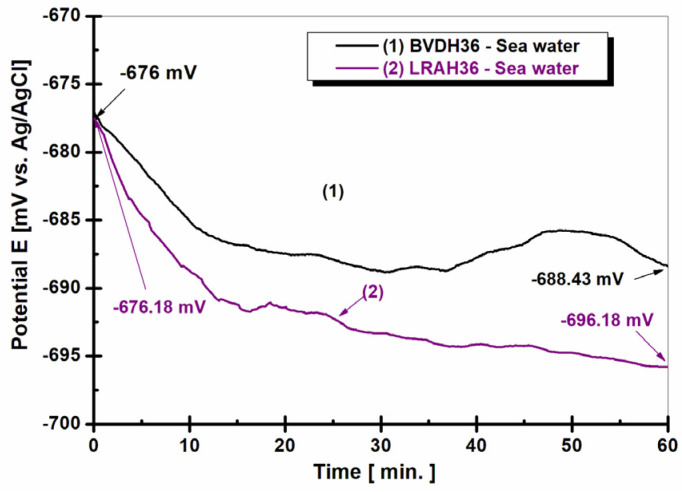
Open circuit potential evolution in seawater during 1 h of immersion: (1) BVDH36 steel; (2) LRAH36 steel.

Figure 1 shows the evolution of the free potential for the two types of studied materials immersed in natural seawater for 60 min.

From Figure 1, it can be seen that both low-alloy steels tend to move the free potential towards negative values; thus, for BVDH36 steel, the value of the initial potential is E = −676 ± 12 mV vs. Ag/AgCl, after one hour reaching the value E = −688.43 ± 21 mV vs. Ag/AgCl. For LRAH36 steel, at immersion it has the value of E = −676.18 ± 35 mV vs. Ag/AgCl, and after one hour of immersion in seawater the value of the free potential is E = −696.18 ± 31 mV vs. Ag/AgCl, at 20 mV vs. Ag/AgCl more negative than the immersion value.

The tendency of the potential to move towards more negative values indicates that there is a dissolution of the oxide layer formed on the surface of the studied materials [3,4,5,6,7,8,9,10], with this behavior leading to a decrease in the corrosion resistance of the materials.

Comparing the seawater immersion behavior of the two steels, it is observed that LRAH36 steel immersed in seawater has a tendency to move towards more negative values, more pronounced compared to BVDH36 steel immersed in seawater. Upon immersion, the value of the free potential for LRAH36 steel immersed in seawater is ∆E = 0.18 mV more negative than the free potential value obtained for BVDH36 steel immersed in seawater. At the end of the 60 min, the difference between the two steels should be a little higher than the value from the immersion. The potential difference between the two steels at the end of the free potential evolution measurement is only 7.75 mV.

### 2.2. Electrochemical Impedance Spectroscopy (EIS) after 1 h of Immersion

The electrochemical impedance spectroscopy (EIS) method is a modern method of studying the processes that take place in an electrochemical cell [9,10]. From an electrical point of view, any cell behaves as an ohmic resistor in a direct current and as an impedance in an alternating current [9,10]. This method consists of disturbing the system with an alternating signal superimposed on the direct current that normally supplies the cell and measuring the response. Thus, the cell can be connected in an alternating current bridge that allows the impedance to be determined [9,10].

**Figure 2:**
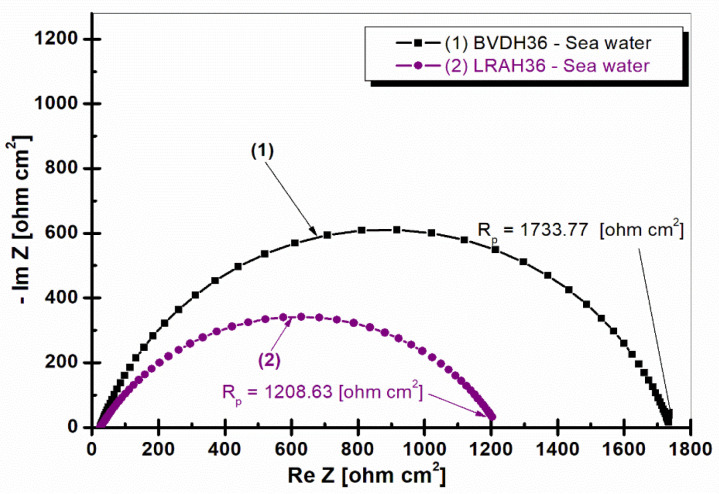
Nyquist plots of electrochemical impedance spectroscopy in seawater after one hour of immersion for (1) BVDH36 steel; (2) LRAH36 steel. Plain symbols are experimental data, plain lines are the fitting results.

Figure 2 shows the Nyquist diagram for the two types of studied steel immersed in natural seawater after one hour’s of immersion.

**Figure 3:**
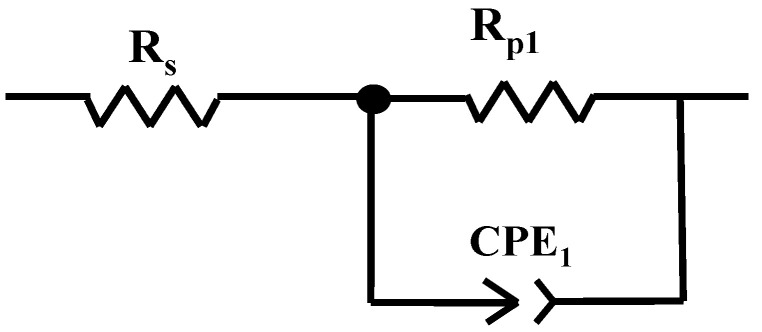
The equivalent electrical circuit used to fit the experimental EIS data.

From Figure 2, it can be seen that BVDH36 steel has a little better polarization resistance, R_p_ = 1733.77 ohm cm^2^, which means a higher corrosion resistance compared to LRAH36, which has a weaker polarization resistance of R_p_ = 1208.63 ohm cm^2^. To obtain the values of the polarization resistance, the experimental data were fitted with the Z_view_ software version 3.4 using a Randles circuit. The equivalent circuit used to fit the experimental data is shown in Figure 3.

The Randles circuit is a simple circuit that is composed of a bias resistor (R_p_), being connected in parallel with the constant phase element (CPE), which replaces the capacity of the ideal capacitor created due to the formation of the electric double layer, in parallel. The circuit is connected in series with the solution resistance (R_s_) representing the resistance of the electrolyte and the resistance of the electrical conductors but also other external resistances of the cell connected in series [9,10].

**Figure 4:**
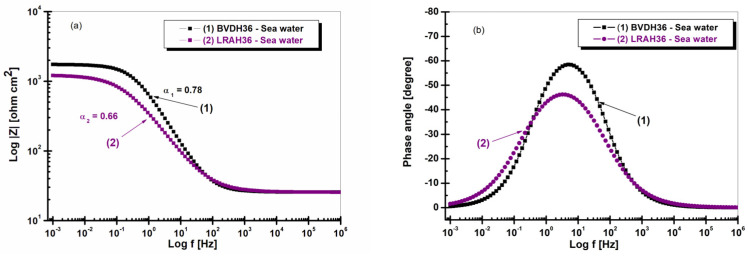
Bode diagram (module Z and phase angle) in seawater after one hour of immersion of (1) BVDH36 steel; (2) LRAH36 steel: (**a**) impedance modulus vs. frequency and (**b**) phase angle vs. frequency.

In the case of the BVDH36 equivalent circuit, the solution resistance is R_s_ = 25.52 ohm. This circuit has a constant phase element, the electrochemical potential capacity, CPE1 = 0.00031058 F/cm^2^, and the angle α_1_ = 0.78 (can be seen in Figure 4a). 

For the LRAH36 equivalent circuit, the solution resistance is R_s_ = 25.48 ohm. The circuit has a constant phase element, namely the electrochemical potential, CPE1 = 0.00076669 F/cm^2^ and the angle α_2_ = 0.66 (can be seen in Figure 4a).

Figure 4a,b show the Bode diagrams of the two low-alloy steels, BVDH36 and LRAH36, immersed in seawater.

Figure 4a has a high impedance value obtained at low frequency, and medium frequency reveals a low reactivity or good corrosion resistance for BVDH36 steel. Figure 4b shows the phase angle as a function of frequency. It can be observed that the maximum value of the phase angle of −63 degrees, which is obtained in the case of BVDH36 steel, compared to LRAH36 steel which has a phase angle of −46 degrees. Figure 4 further demonstrates that the corrosion resistance of the BVDH36 steel sample is better as compared to the LRAH36 steel sample.

### 2.3. Evolution of Open Circuit Potential in Seawater for 26 h of LRAH36 Steel and BVDH36 Steel

**Figure 5:**
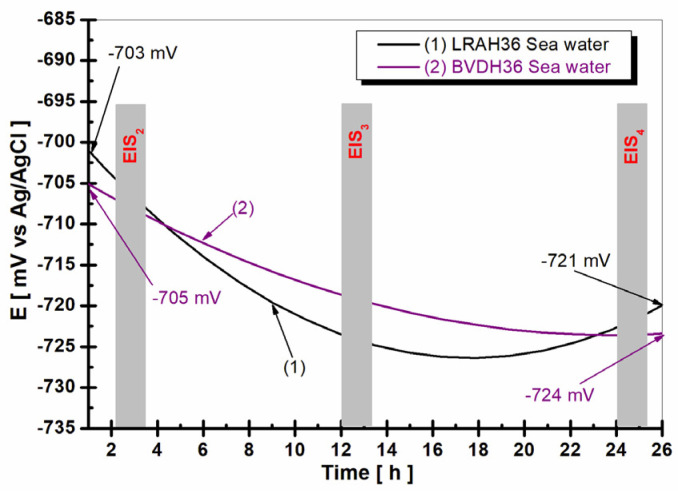
Open circuit potential evolution in seawater during 26 h of (1) BVDH36 steel; (2) LRAH36 steel. The bars show the time of impedance spectroscopy measurements.

Figure 5 shows the evolution of the free potential of BVDH36 and LRAH36 steels immersed in natural seawater for a period of 26 h.

Analyzing the data in Figure 5, it can be seen that the open circuit potential trend during the 26 h of immersion the BVDH36 steel has a less negative trend than LRAH36. As seen at the beginning of the corrosion test, the initial dip value is E = −705 ± 12 mV vs. Ag/AgCl, reaching the value E = −724 ± 15 mV vs. Ag/AgCl.

In the case of LRAH36 steel, the open circuit potential during the 26 h moves towards more negative values, starting from the initial potential E = −703 ± 33 mV vs. Ag/AgCl and reaching after 26 h the value E = −721 ± 28 mV vs. Ag/AgCl.

The continuous negative trend of the open circuit potential during the 26 h of immersion for both low-alloy steels BVDH36 and LRAH36 is attributed to the inability of the steels to retain corrosion products on their surface, which could function as protective layers of oxides.

### 2.4. Electrochemical Impedance Spectroscopy (EIS) after 3.3 h, 12 h, and 24.3 h of Immersion in Seawater of LRAH36 and BVDH36 Steel Samples

**Figure 6:**
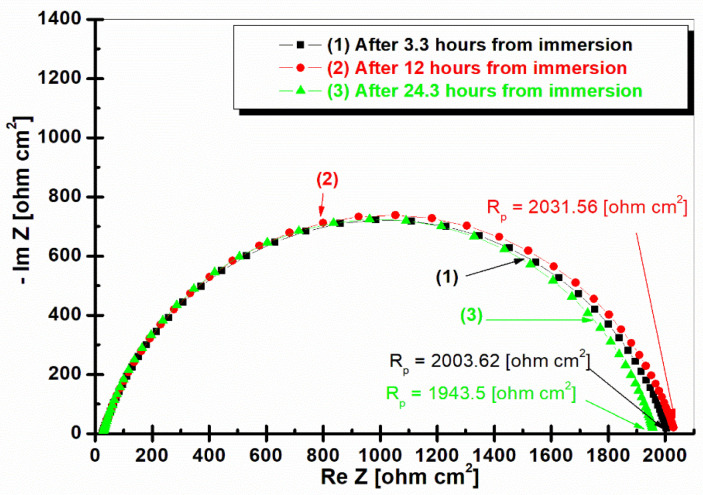
Nyquist plots of electrochemical impedance spectroscopy of BVDH36 steel in seawater after 3.3 h of immersion, after 12 h of immersion, and after 24.3 h of immersion. The simple symbols are the experimental data; the simple lines are the fitting results.

Figure 6 shows electrochemical impedance spectroscopy in the form of a Nyquist representation for BVDH36 steel immersed in natural seawater at different time intervals.

From Figure 6, it can be seen that in the superimposed Nyquist impedances from BVDH36 steel the polarization resistance increases and finally decreases. So that after 3.3 h of immersion it has a polarization resistance of 2003.62 ohm cm^2^. After 12 h of immersion the polarization resistance value is a little higher being 2031.56 ohm cm^2^. The value decreases after 24.3 h of immersion in seawater, having the polarization resistance of 1943.5 ohm cm^2^.

**Figure 7:**
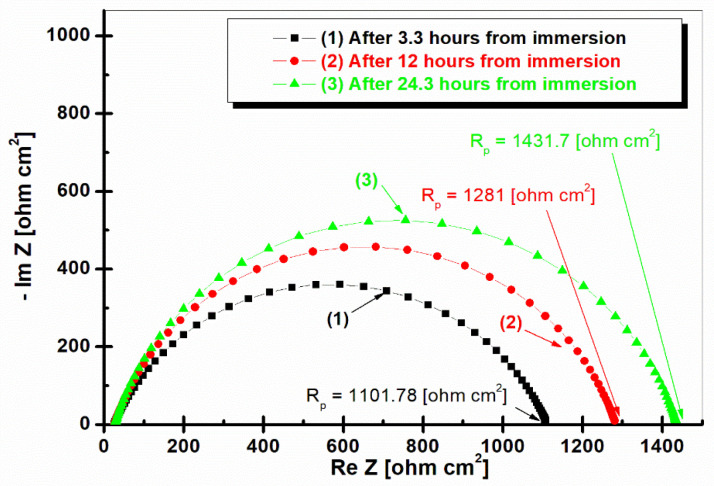
Nyquist plots of electrochemical impedance spectroscopy of LRAH36 steel in seawater after 3.3 h of immersion, after 12 h of immersion, and after 24.3 h of immersion. The simple symbol represents experimental data; the simple line is the fitting result.

Figure 7 shows the electrochemical impedance spectroscopy in the form of a Nyquist representation for LRAH36 steel immersed in natural seawater at different time intervals.

From Figure 7, it can be seen that the polarization resistance increases over time, along with the number of impedances; thus, after 3.3 h of immersion it has the lowest polarization resistance of 1101.78 ohm cm^2^, reaching after 24.3 h of immersion in seawater the highest polarization resistance at 1431.7 ohm cm^2^.

Comparing Figure 6 and Figure 7, it can be seen that BVDH36 steel has a higher polarization resistance, compared to LRAH36 steel, which has a lower polarization resistance, that is, a weaker corrosion resistance.

**Figure 8:**
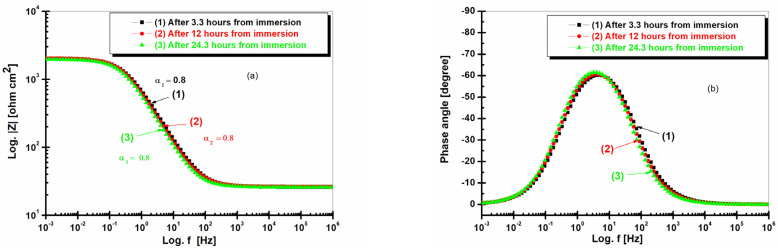
Evolution of electrochemical impedance spectroscopy in Bode representations for BVDH36 steel immersed in seawater after 3.3 h of immersion, after 12 h of immersion, and after 24.3 h of immersion: (**a**) Modul Z vs. frequency and (**b**) phase angle vs. frequency.

Figure 8 shows the electrochemical impedance spectroscopy as a Bode representation for BVDH36 steel immersed in natural seawater at different time intervals.

From Figure 8a, it can be seen that BVDH36 steel with Bode diagrams after 3.3 h of immersion, after 12 h of immersion and after 24.3 h of immersion in seawater, respectively, has an impedance well above 10^3^ ohm cm^2^ between the frequencies 10^−^^3^ and 10^−^^1.2^ Hz, and the impedance decreases after 3.3 h of immersion, after 12 h of immersion, and after 24.3 h of immersion, being 10 ohm cm^2^ between the frequencies of 10^2^ and 10^6^ Hz. The angles for Bode diagrams after 3.3 h, 12 h, and 24.3 h of immersion are the same with α1 = α2 = α3 = 0.8. From Figure 8b of BVDH36 steel, it can be seen that in the Bode diagrams after 3.3 h of immersion, after 12 h of immersion, and after 24.3 h of immersion, respectively, the phase angle attains the value of −67 degrees.

**Figure 9:**
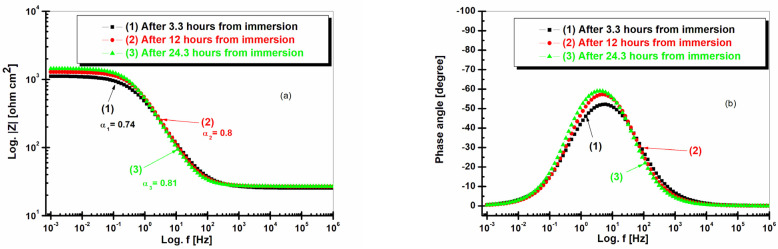
Evolution of electrochemical impedance spectroscopy in Bode representations for LRAH36 steel immersed in seawater after 3.3 h of immersion, after 12 h of immersion, and after 24.3 h of immersion: (**a**) Modul Z vs. frequency and (**b**) phase angle vs. frequency.

Figure 9 shows the electrochemical impedance spectroscopy in the form of a Bode representation for LRAH36 steel immersed in natural seawater at different time intervals.

In Figure 9a, LRAH36 steel in Bode diagrams after 3.3 h of immersion, after 12 h of immersion, and after 24.3 h of immersion in seawater has maximum impedances slightly above 10^3^ ohm cm^2^ between the frequencies of 10^−^^3^ and 10^−^^1.3^ Hz, and the impedance drops to 10 ohm cm^2^ between the frequencies of 10^2^ and 10^6^ Hz. From Figure 9a, LRAH36 steel has maximum impedances slightly above 10^3^ ohm cm^2^, after 3.3 h of immersion, after 12 h of immersion, and after 24.3 h of immersion, respectively, which leads to the conclusion that BVDH36 steel is a little more resistant to corrosion than LRAH36 steel, where BVDH36 steel, from Figure 8a of the Bode diagrams after 3.3 h of immersion, after 12 h of immersion, and after 24.3 h of immersion, respectively, can be seen to have a maximum impedance well above 10^3^ ohm cm^2^. The depressed angles are observed in Figure 9a, with α_1_ = 0.74 after 3.3 h of immersion, α2 = 0.8 after 12 h of immersion, and α3 = 0.81 after 24.3 h of immersion.

From Figure 9b, it can be seen that LRAH36 steel after 3.3 h of immersion has a maximum phase angle of −53 degrees, after 12 h of immersion has a phase angle of approximately −59 degrees, and after 24.3 h of immersion has a phase angle of −63 degrees.

### 2.5. Polarization Resistance and Corrosion Rate

**Figure 10:**
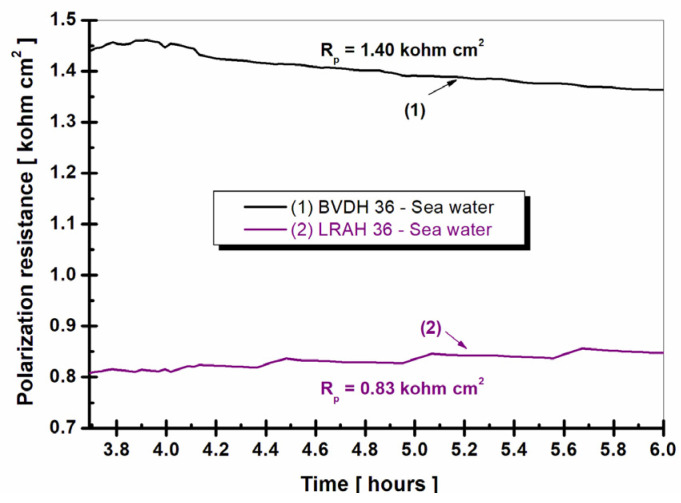
Evolution of polarization resistance of BVDH36 and LRAH36 steel immersed in seawater.

Figure 10 shows the evolution over time of the polarization resistance in natural seawater of BVDH36 and LRAH36 steels.

Polarization resistance and corrosion rate are evaluated by the linear polarization method by plotting 100 linear polarization curves. 

Using the 100 polarization curves by plotting the Tafel slopes with Stern Geary equation [3], as the Equations (1) and (2), 100 values of polarization resistances and 100 values of corrosion rate were calculated for each alloy on each measurement step.

(1) $$R_{p}=\frac{B}{i_{corr}}=\frac{\left(\Delta E\right)}{{\left(\Delta i\right)}_{\Delta E\mapsto 0}}$$

(2) $$B=\frac{b_{a}\cdot b_{c}}{2.3(b_{a}+b_{c})}$$

where

*R_p_* is the polarization resistance;

*i_corr_* the corrosion current density;

The proportionality constant, *B*, for a particular system can be determined as shown by Stern and Geary, calculated from *b_a_* and *b_c,_* the slopes of the anodic and cathodic Tafel, Equation (2).

From the analysis of Figure 10, it can be seen that the average value of the polarization resistance in the case of BVDH36 steel is approximately two times higher than the value obtained by LRAH36 steel. At the same time, it is well known in the specialized literature that the polarization resistance is inversely proportional to the corrosion rate; in other words, the higher the polarization resistance for a studied material, the lower the corrosion rate.

**Figure 11:**
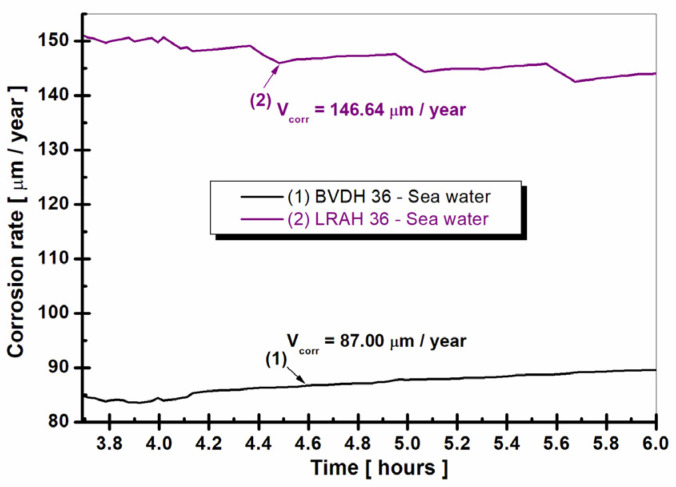
Evolution of corrosion rate as penetration rate of BVDH36 and LRAH36 steel immersed in seawater.

Figure 11 shows the evolution over time of the corrosion rate as the penetration rate into natural seawater of BVDH36 and LRAH36 steel.

From the analysis of Figure 11, it can be seen that the average value of the corrosion rate in the case of BVDH36 steel is approximately two times lower than the value obtained by LRAH36 steel. If for BVDH36 steel we have a value of V_corr_ = 87 µm/year, for LRAH36 steel we have a value of V_corr_ = 146.64 µm/year, thus indicating that BVDH36 steel has a higher corrosion resistance compared to LRAH36 steel immersed in natural seawater.

### 2.6. Potentiodynamic Polarization Plots, Logarithmically Scaled for Current Density, Obtained in Seawater for Steels BVDH36 and LRAH36 (Tafel Representation)

**Figure 12:**
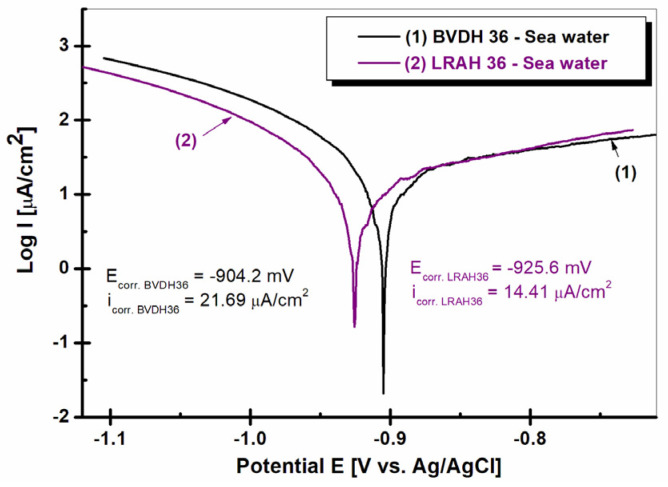
Potentiodynamic polarization plots, logarithmically scaled for current density, obtained in seawater for steels BVDH36 and LRAH36 (Tafel representation).

Figure 12 shows the Tafel curves for BVDH36 and LRAH36 steels immersed in natural seawater.

**Figure 13:**
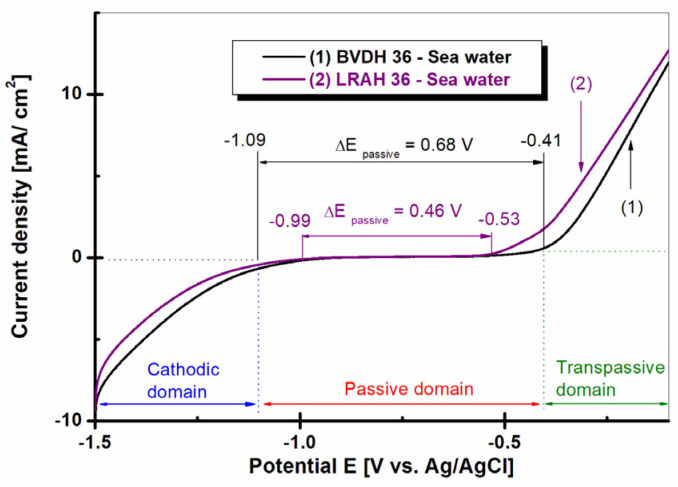
Potentiodynamic polarization diagrams (PD1) were recorded in the potential domain from −1.5 V to 1 V vs. Ag/AgCl, with a scan rate of 1 mV/s in seawater for (1) BVDH36 steel and (2) LRAH36 steel.

As can be seen from the polarization diagrams shown in Figure 13, the corrosion potentials show a shift towards negative values for both low-alloy steels. The lowest value of the E_corr_ corrosion potential was recorded in the case of BVDH36 steel (−904.2 mV (vs. Ag/AgCl)), which indicates that BVDH36 steel is slightly more resistant in seawater compared to LRAH36 steel (−925.6 mV (vs. Ag/AgCl)). The systematic evolution is in the following order: E_corr_: BVDH36 < LRAH36; and for i_corr_: BVDH36 (21.69 µA/cm^2^) < LRAH36 (14.41 µA/cm^2^). In general, the more positive the E_corr_ value is, the more susceptible the material is to corrosion processes.

In order to investigate the effect of seawater on the passive range of BVDH36 and LRAH36 steel immersed in natural seawater, the potentiodynamic polarization curves were drawn, the results of which are presented in Figure 13.

As shown in Figure 13, curve (1), BVDH36 steel immersed in natural seawater shows a wide range of passivation between E_1_ = −1.09 V vs. Ag/AgCl and E_2_ = −0.41 V vs. Ag/AgCl, having a passivation range of ΔEpassiv = 0.68 V. The LRAH36 steel curve (2) immersed in natural seawater has a smaller passivation range compared to BVDH36 steel immersed in seawater which is between E_3_ = −0.99 V vs. Ag/AgCl and E4 = −0.53 V vs. Ag/AgCl, having a passivation range of ΔE_passiv_ = 0.46 V. The wider the passivation range of a material, the better the corrosion behavior.

### 2.7. Cyclic Voltammetry

**Figure 14:**
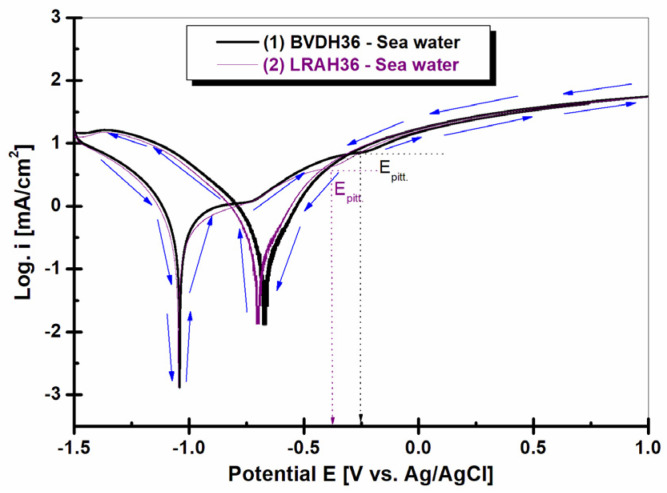
The cyclic voltammetry curve (CV) was recorded in the potential range starting from −1.5 V, returning 1 V, and ending at −1.5 V vs. Ag/AgCl, with a scanning speed of 5 mV/s in seawater of (1) BVDH36 steel and (2) LRAH36 steel. Logarithmic scale representation for current density and pitting potential determination. The arrows indicate the scanning potential direction.

Figure 14 shows the cyclic voltammetry for BVDH36 and LRAH36 steels immersed in natural seawater.

From Figure 14, at the intersection of the inflection point, where the current density suddenly increases and changes its slope, the pitting corrosion potential of BVDH36 steel is E_pit_ = −0.25 V and LRAH36 steel has the pitting corrosion potential E_pit_ = −0.37 V. The more positive the pitting corrosion potential is, the more resistant it is to corrosion.

### 2.8. Optical Microscopy of BVDH36 and LRAH36 Steels before and after Corrosion

**Figure 15:**
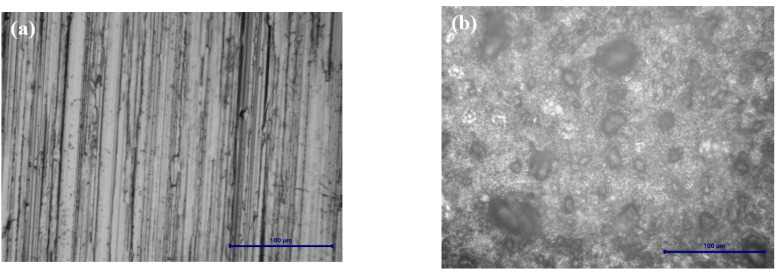
Optical microscopy images at magnification scale (50×) for the steel BVDH36: (**a**) before corrosion and (**b**) after corrosion.

Figure 15 shows the optical microscopy for BVDH36 steel before and after the corrosion process.

**Figure 16:**
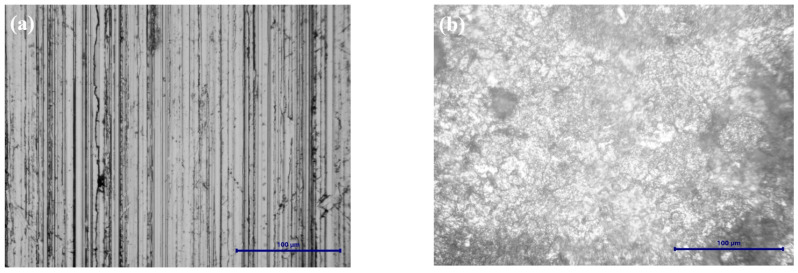
Optical microscopy images at magnification scale (50×) for the steel LRAH36: (**a**) before corrosion and (**b**) after corrosion.

Figure 16 shows the optical microscopy for LRAH36 steel before and after corrosion.

The optical microscopy images, presented in Figure 15b and Figure 16b, after corrosion highlight the occurrence of pitting corrosion being much more pronounced in the case of LRAH36 steel compared to BVDH36 steel.

### 2.9. Morphological and Compositional Characterization of the Surfaces of BVDH36 and LRAH36 Steels before and after Corrosion by Scanning Electron Microscopy (SEM-EDX) 

**Figure 17:**
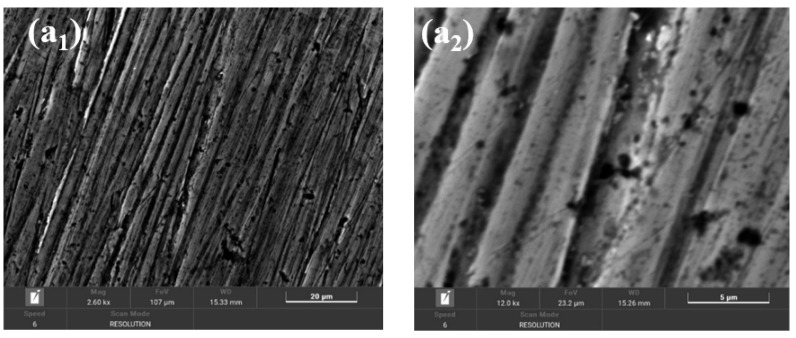

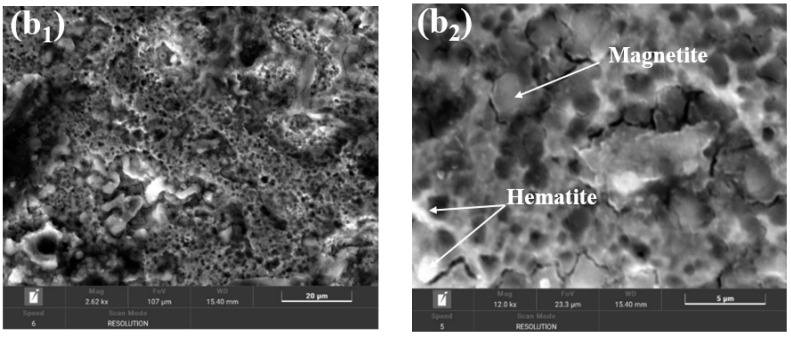
SEM morphology for sample BVDH 36: (**a_1_**,**a_2_**) before corrosion, (**b_1_**,**b_2_**) after corrosion immersed in natural seawater.

Figure 17 shows the SEM images of BVDH36 steel before (Figure 17a_1_,a_2_) and after the corrosion process (Figure 17b_1_,b_2_).

From Figure 17a_2_ it can be seen that BVDH36 steel before corrosion has a clean surface, without corrosion products, but with slight surface defects due to mechanical processing of surfaces. After the corrosion, the appearance of raised agglomerated formations associated with the corrosion products formed on the surface of BVDH36 steel immersed in natural seawater (Năvodari area) can be observed. Using the data from the specialized literature, from Figure 17b_2_ it is possible to identify hematite (Fe_2_O_3_) in the form of small spherical crystalline globules [19] and the appearance of dark flat surfaces of magnetite (Fe_3_O_4_) [20]. The formation of corrosion products is also confirmed by the data obtained from energy-dispersive X-ray spectroscopy (EDX) by the appearance of the element oxygen (Table 1).

From Table 1, of the EDX spectrum, for the BVDH36 sample, before corrosion we have the following mass fractions % by weight (Wt %): 90.2% Fe; 6.6% C; 1.7% Mn; 1.2% Si; 0.1% Ni; 0.1% P; and 0.1% Al, and atomic fractions (σ %): 1.3% Fe; 1.3% C; 0.1% Mn; 0.1% Si; 0.1% Ni; 0.1% P; and 0.1% Al.

From Table 2, of the EDX spectrum, for the BVDH36 sample, after corrosion we have the following mass fractions % by weight (Wt %): 68.5% Fe; 15.6% C; 14.8% O; 0.9% Mn; 0.1% Ni; and 0.1% Cu, and the corresponding atomic fractions (σ %): 0.6% Fe; 0.6% C; 0.3% O; 0.1% Mn; 0.1% Ni; and 01% Cu.

Comparing the data obtained from the two EDX spectra of the BVDH36 sample before corrosion (Table 1) and after corrosion (Table 2), it can be seen that after corrosion, the percentage of oxygen increases to the value of 14.3 Wt%, while the percentage of iron decreases from 90.2 Wt% before corrosion to 68.5 Wt% after corrosion, which confirms the appearance of some oxides on the surface of the sample.

**Figure 18:**
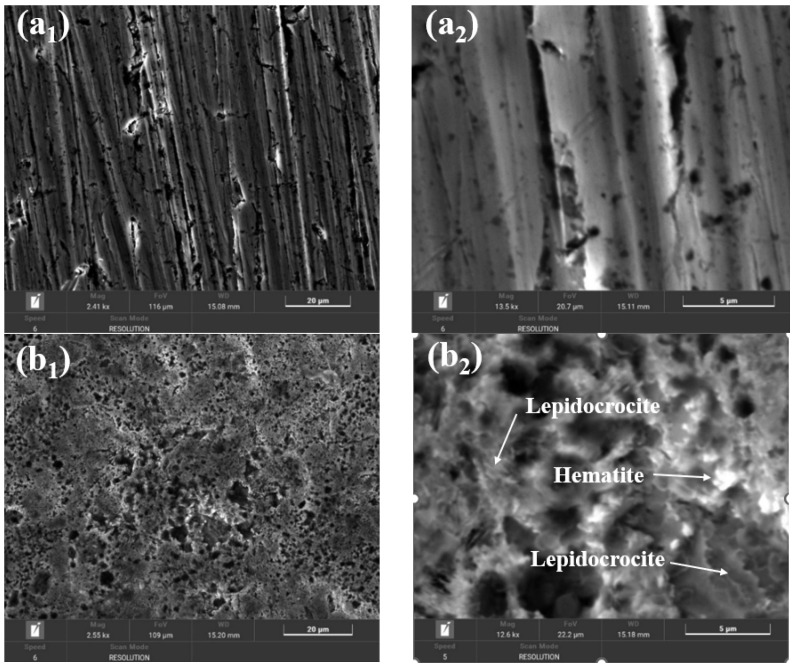
SEM morphology for sample LRAH36: (**a_1_**,**a_2_**) before corrosion, (**b_1_**,**b_2_**) after corrosion immersed in natural seawater.

The SEM morphology of the surface of the LRAH36 steel samples before and after corrosion is shown in Figure 18. As with the BVDH36 sample (Figure 17a_1_,a_2_), in Figure 18a_1_,a_2_ it can be seen that the surface of the LRAH36 steel before corrosion is clean, without corrosion products, but with slight surface defects due to mechanical processing activities. Comparing the morphologies of the two low-alloy steels after corrosion (Figure 17b_1_,b_2_) with Figure 18b_1_,b_2_, it is observed that the surface of the LRAH36 steel is covered by an almost uniform layer of corrosion products, a layer which covers the entire surface of the steel. The formation of corrosion products is also confirmed by the data obtained from the EDX spectrum, by the appearance of the oxygen element (Table 3).

Based on the data from the specialty literature regarding the analysis of the surface morphology of the corrosion products [19,20], the following corrosion products were identified: hematite in the form of small spherical crystalline globules and lepidocrocite with a laminar “bird’s nest” appearance and lepidocrocite in the form of flower petals.

The results obtained by the energy-dispersive X-ray spectroscopy (EDX) technique are shown in Table 3 and Table 4.

From Table 3, of the EDX spectrum, for sample LRAH36, before corrosion, we have the following mass fractions % by weight (Wt %): 91.5 Fe; 6% C; 1.6% Mn; 0.5% Si; 0.2% Mo; 0.1% Ni; and 0.1% Al, and atomic fractions (σ %): 1.3% Fe; 1.2% C; 0.1% Mn; 0.1% Si; 0.3% Mo; 0.1% Ni; and 0.1% Al.

From Table 4, of the EDX spectrum, for sample LRAH36, after corrosion we have the following mass fractions % by weight (Wt %): 80.7% Fe; 10.8% C; 6.4% O; 1.2% Mn; 0.3% Cu; 0.3% Si; 0.1% Ni; 0.1% Al; and 0.1% P, and corresponding atomic fractions (σ %): 0.7% Fe; 0.8% C; 0.3% O; 0.1 Mn; 0.1% Cu; 0.1% Si; 0.1% Ni; 0.1% Al; and 0.1% P. From the comparative analysis of the two EDX spectra (Table 3 and Table 4) corresponding to the LRAH36 low-alloy steel samples, it was found that in the case of the LRAH36 sample after corrosion, the percentage of oxygen increases to a value of 6.4 Wt%, while the percentage of iron decreases from a value of 91.5 Wt% before corrosion (Table 3) to a value of 80.7 Wt% after corrosion (Table 4). 

### 2.10. Structural Analysis of BVDH36 and LRAH36 Steels before and after Corrosion Using the XRD Technique

**Figure 19:**
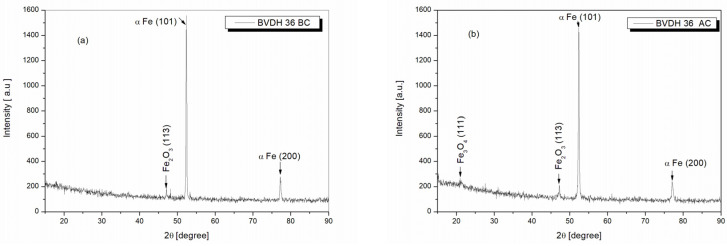
X-ray diffractograms of BVDH36 samples: (**a**) before corrosion, (**b**) after corrosion immersed in natural seawater.

Figure 19a,b show the X-ray diffractograms of BVDH36 steel samples before corrosion and after corrosion. Analyzing the ray diffractogram in Figure 19a, it was observed that for BVDH36 steel before corrosion, the dominant diffraction peak corresponds to the (101) plane identified at the 2θ angle of 52.35°. This diffraction peak belongs to the element α-Fe. Also, α-Fe was identified at the angle 2θ of 77.20°, but with the intensity of the diffraction peak corresponding to the lower (200) plane. The element α-Fe with the crystallographic planes (101 and 200) was identified using the card COD 96-110-0109. Next to the element α-Fe, identified using the card COD 96-210-8029, the compound Fe_2_O_3_ was observed at the angle 2θ of 47.20° with the crystallographic plane (113), but with the intensity of the diffraction peak being much lower than the intensities of the diffraction peaks belonging to the element α-Fe.

From the analysis of the X-ray diffractogram of BVDH36 steel after corrosion shown in Figure 19b, it is observed that the diffraction peaks identified before corrosion are preserved, respectively, for the element α-Fe and compound Fe_2_O_3_ (hematite) at the same 2θ diffraction angles with the same crystallographic planes. However, compared with the BVDH36 steel sample before corrosion, in the case of BVDH36 steel after corrosion, it can be seen that the intensities of the two diffraction peaks corresponding to the element α-Fe are lower, while the intensity of the hematite diffraction peak Fe_2_O_3_ increases 1.6 times, and in addition the compound Fe_3_O_4_ (magnetite) appears at the 2θ angle of 21.44° related to the crystallographic plane (111) identified with the card COD 96-900-2321. The results obtained from the XRD data (Figure 19) are in good agreement with those obtained from SEM-EDX images.

Table 5 shows the centralized data obtained by the X-ray diffraction technique of the two low-alloy steels BVDH36 and LRAH36, before corrosion and after corrosion in natural seawater.

**Figure 20:**
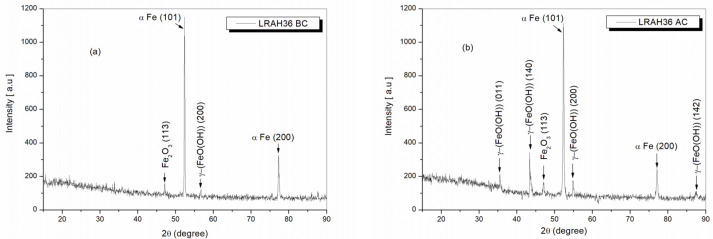
X-ray diffractograms of LRAH36 samples: (**a**) before corrosion, (**b**) after corrosion immersed in natural seawater.

Figure 20 shows the X-ray diffractograms of the LRAH36 steel samples before corrosion (Figure 20a) and after corrosion (Figure 20b).

From Figure 20a, in the case of LRAH36 steel before corrosion, the following crystalline phases were identified: the compound Fe_2_O_3_ at the angle of 47.20° with the crystallization plane (113) using the card COD 96-210-8029; the element α-Fe with planes (101) and (200) using COD 96-110-0109. Also, with the help of the card COD 96-901-5157, the compound γ-FeO(OH) (lepidocrocite) was also identified at the 2θ angle of 54.97°, having the crystallographic plane (200).

From the analysis of the X-ray diffraction spectrum in Figure 20b, it is observed that in the case of the LRAH36 sample after corrosion, the diffraction peaks identified before corrosion on the same type of sample studied show, respectively, the elements α-Fe and hematite Fe_2_O_3_ at the same 2θ diffraction angles and with the same crystallographic planes.

Also, as in the case of BVDH36 steel after corrosion, it can be seen that the intensities of the two diffraction peaks corresponding to the element α-Fe are lower, while the intensity of the diffraction peak of hematite increased 1.8 times. It should be noted that in addition to the sample studied before corrosion (Figure 20a), in the case of this sample (Figure 20b), the corrosion product, lepidocrocite, appears as γ-(FeO(OH)) at the following diffraction angles 2θ: 35.53°, 43.70°, 54.97°, and 87.58°, with the crystallographic planes (011, 140, 200, 142) identified with the card COD 96-901-5157.

The results obtained from the XRD investigation (Figure 20) are in good agreement with those obtained from the SEM-EDX images, confirming that the rust layer in the case of LRAH36 steel consists of the lepidocrocite compound γ-FeO(OH) and hematite Fe_2_O_3_, with the observation that the corrosion product lepidocrocite is in a greater proportion than hematite.

## 3. Materials and Methods

### 3.1. Materials

For this study, two types of low-alloy steels in the naval field (BVDH36 and LRAH36) purchased from the Galati (Liberty, Galati, Romania) steel plant were used. The composition of the two steels is shown in Table 6 and Table 7.

To carry out the electrochemical tests, the steel samples were cut to the size of 2.5 cm × 2.5 cm × 3 mm, glued with a copper wire to have electrical contact, and embedded in epoxy resin to be able to have a well-defined active surface: the steel BVDH36 had an area of 2.52 cm^2^ and the steel LRAH36 had an area of 2.55 cm^2^. Before each experiment, the samples were sanded with SiC 1200# abrasive paper to remove the oxide layer formed on their surface, then cleaned and rinsed with distilled water.

### 3.2. Electrochemical Test

The PGZ 100 electrochemical equipment was used, on which the VoltaMaster 4 software version 5.1 was running. The electrochemical tests were performed at room temperature, 25 ± 1 °C, in an electrochemical cell composed of three electrodes. The working electrode (WE) is the tested steel sample of BVDH36 and LRAH36. The counter electrode (CE) is formed by a Pt-Rh grid, and the reference electrode (RE) is Ag/AgCl with saturated KCl solution that has a potential of +199 mV vs. NHE. The volume of electrolyte used for each experiment was 150 mL (natural seawater from the Black Sea, Năvodari, România). The physicochemical characteristics of natural seawater measured before the electrochemical tests using a multiparameter Consort C533 are presented in Table 8.

**Figure 21:**
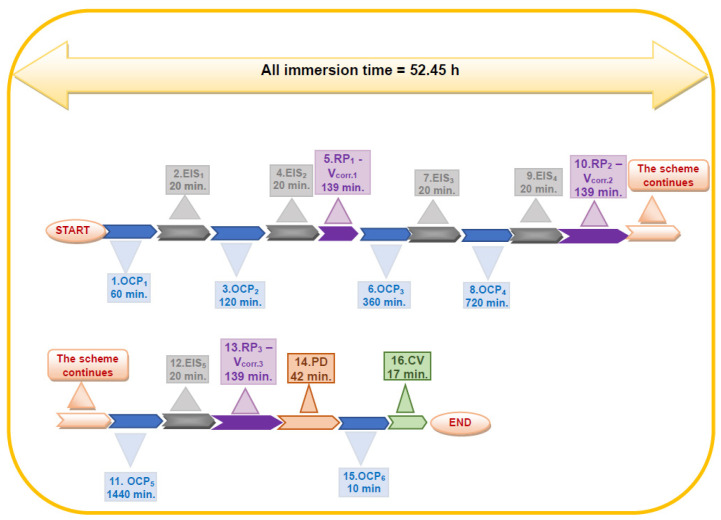
Schematic protocol for electrochemical measurements for corrosion evaluation of low-alloy steels: BVDH36 and LRAH36.

To determine the corrosion resistance of low-alloy steels immersed in natural seawater, the following experimental protocol is applied (Figure 21).

1. OCP_1_—Open circuit potential 1, 4 s measurement period, 1 h duration. 

2. EIS_1_—Electrochemical Impedance Spectroscopy 1—Frequency from 100 kHz to 100 mHz, AC = 10 mV, Freq./decade 20, duration 20 min.

3. OCP_2_—Open circuit potential 2, 4 s measurement period, 2 h duration.

4. EIS_2_—Electrochemical Impedance Spectroscopy 2—Frequency from 100 kHz to 100 mHz, AC = 10 mV, Freq./decade 20, duration 20 min.

5. Rp_1_—Vcor_1_–Bias resistance 1—Corrosion rate 1: ±40 mV, 100 points, 5 mV/s, duration 139 min.

6. OCP_3_—Open circuit potential 3, 4 s measurement period, 6 h duration.

7. EIS_3_– Electrochemical Impedance Spectroscopy 3—Frequency from 100 kHz to 100 mHz, AC = 10 mV, Freq./decade 20, duration 20 min.

8. OCP_4_—Open circuit potential 4, 4 s measurement period, 12 h duration.

9. EIS_4_—Electrochemical Impedance Spectroscopy 4—Frequency from 100 kHz to 100 mHz, AC = 10 mV, Freq./decade 20, duration 20 min.

10. Rp_2_—Vcor_2_–Polarization resistance 2—Corrosion rate 2: ±40 mV, 100 points, 5 mV/s, duration 139 min.

11. OCP_5_—Open circuit potential 5, 4 s measurement period, 24 h duration.

12. EIS5—Electrochemical Impedance Spectroscopy 5—Frequency from 100 kHz to 100 mHz, AC = 10 mV, Freq./decade 20, duration 20 min.

13. Rp_3_—Vcor_3_–Polarization resistance 3—Corrosion rate 3: ±40 mV, 100 points, 5 mV/s, duration 139 min.

14. PD—Potentiodynamic bias 1—from −1.5 V to 1.0 V; scan rate: 1 mV/s, sampling 1:4, duration 42 min.

15. OCP_6_—Open circuit potential 6, measurement period 4 s, duration 10 min.

16. CV—Cyclic voltammetry—from −1.5 V to 1.0 V to −1.5 V, scan speed: 5 mV/s, 1 cycle, duration 17 min.

The experimental results are presented using the Origin 2022 software version 9.9. Each experiment was repeated three times in order to check the reproducibility of the experimental results.

### 3.3. Characterization of Low-Alloy Steels before and after Electrochemical Tests

An OPTIKA XDS 3MET metallurgical optical microscope was used to highlight the surface morphology of the studied steels.

In order to obtain high-resolution detailed images of BVDH36 and LRAH36 steel samples before and after corrosion, the TESCAN scanning electron microscope was used. This scanning electron microscope is equipped with an EDX detector. Energy-dispersive X-ray spectroscopy (EDX) was used in this study to determine the elemental chemical composition. EDX data were processed using Bruker Esprit software v2.2, which enables easy EDX elemental chemical composition analysis and sample mapping.

In order to identify the crystalline phases of the studied steels, the X-ray diffraction technique was applied. Based on X-ray diffraction analysis recorded with Drone Test Equipment 3 using a Cobalt anode ( ), on the field between 15 and 90°, diffraction spectra were obtained, which were later matched using the standardized data of Match!3 software version 4.0 for phase identification (http://www.crystalimpact.com/match accessed on 6 March 2024), and the results were matched to the crystallographic database (Crystallography Open Database, COD), identifiable with a 9-digit code.

## 4. Conclusions

In this work, the corrosion behavior in Black Sea water of two low-alloy steels, BVDH36 and LRAH36, was studied for 52.45 h by electrochemical methods.

Comparing the seawater immersion behavior of the two steels, it is observed that LRAH36 steel immersed in seawater tends to move towards more negative, more pronounced values than BVDH36 steel immersed in seawater. During immersion, the free potential value for LRAH36 steel immersed in seawater is 0.18 mV vs. Ag/AgCl more negative than the free potential value obtained for BVDH36 steel immersed in seawater. After 60 min, the difference between the two steels is slightly greater than the value at immersion. Basically, the potential difference between the two steels at the end of the free potential evolution measurement is 7.75 mV compared to Ag/AgCl. The tendency of the potential to move towards more negative values indicates that there is a dissolution of the oxide layer formed on the surface of the studied materials, this behavior leading to a decrease in the corrosion resistance of the materials.

The continued trend towards negative values of the open circuit potential over 26 h of the low-alloy steels BVDH36 and LRAH36 is attributed to the inability of the steels to retain corrosion products on their surface, which could act as protective oxide layers.

From the plots in the Nyquist plot, it can be seen that LRAH36 steel is slightly weaker in corrosion due to its lower polarization resistance (EIS after 1 h of immersion has a polarization resistance of 1208.63 ohm cm^2^, with EIS after 24.3 h of immersion reaching a polarization resistance of 1431.7 ohm cm^2^), compared to BVDH36 steel, which has a higher corrosion resistance due to the higher value of polarization resistance (EIS after 1 h of immersion: polarization resistance of 1733.77 ohm cm^2^, with EIS after 12 h of immersion reaching the highest polarization resistance at 2031.56 ohm cm^2^).

From the Bode plots, it was observed that BVDH36 steel is slightly more resistant to corrosion as it has a maximum impedance of well above 10^3^ ohm cm^2^ between the frequencies of 10^−3^ and 10^−1.2^ Hz and has a phase angle of about −67 degrees, compared with LRAH36 steel, which has a maximum impedance of just over 10^3^ ohm cm^2^ and a phase angle of −63 degrees.

In potentiodynamic polarization diagrams, PD, BVDH36 steel has a higher passivation range than LRAH36 steel, which indicates that BVDH36 steel is more corrosion-resistant than LRAH36. 

Corrosion in pitting is very dangerous because the pittings have small diameters, being hardly visible, but they are deep and can affect the structure of the steel. Based on the results obtained from the cyclic voltammetry, the more pronounced sensitivity of LRAH36 steel to pitting corrosion in seawater was highlighted compared to BVDH36 steel. 

The results obtained from the SEM-EDX investigations are in good agreement with those obtained from the XRD data. Thus, for both samples of low-alloy steels before corrosion, BVDH36 and LRAH36, their surface is clean, without corrosion products, but with slight surface defects due to mechanical processing activities. In the case of BVDH36 steel after corrosion, the formed corrosion products are hematite Fe_2_O_3_ and magnetite Fe_3_O_4_. In the case of LRAH36 steel after corrosion, the entire surface of the steel is covered by an almost uniform layer of corrosion products, which consists of the compound lepidocrocite γ-FeO(OH) and hematite Fe_2_O_3_, with the observation that the corrosion product lepidocrocite is in a greater proportion than hematite.

## Figures and Tables

**Table 1:** EDX spectra for BVDH36 before corrosion.

Chemical Elements	Wt %	σ %
Fe	90.2%	1.3%
C	6.6%	1.3%
Mn	1.7%	0.1%
Si	1.2%	0.1%
Ni	0.1%	0.1%
P	0.1%	0.1%
Al	0.1%	0.1%

**Table 2:** EDX spectra for BVDH36 after corrosion.

Chemical Elements	Wt %	σ %
Fe	68.5%	0.6%
C	15.6%	0.6%
O	14.8%	0.3%
Mn	0.9%	0.1%
Ni	0.1%	0.1%
Cu	0.1%	0.1%

**Table 3:** EDX spectra for LRAH36 before corrosion.

Chemical Elements	Wt %	σ %
Fe	91.5%	1.3%
C	6%	1.2%
Mn	1.6%	0.1%
Si	0.5%	0.1%
Mo	0.2%	0.3%
Ni	0.1%	0.1%
Al	0.1%	0.1%

**Table 4:** EDX spectra of chemical elements for LRAH36 after corrosion.

Chemical Elements	Wt %	σ %
Fe	80.7%	0.7%
C	10.8%	0.8%
O	6.4%	0.3%
Mn	1.2%	0.1%
Cu	0.3%	0.1%
Si	0.3%	0.1%
Ni	0.1%	0.1%
Al	0.1%	0.1%
P	0.1%	0.1%

**Table 5:** Information obtained by the X-ray diffraction technique of the two low-alloy steels BVDH36 and LRAH36, before corrosion and after corrosion in natural seawater.

Type of Sample	2θ	Intensity Peak	Miller Indices (hkl)	Crystallographic Phases	COD (Crystallography Open Database)
BVDH36 before corrosion	47.20°	130	(113)	Fe_2_O_3_ (hematite)	COD 96-210-8029
	52.35°	1557	(101)	α Fe	COD 96-110-0109
	77.20°	278	(200)	α Fe	COD 96-110-0109
BVDH36 after corrosion	21.44°	232	(111)	Fe_3_O_4_ (magnetite)	COD 96-900-2321
	47.20°	209	(113)	Fe_2_O_3_ (hematite)	COD 96-210-8029
	52.35°	1458	(101)	α Fe	COD 96-110-0109
	77.20°	200	(200)	α Fe	COD 96-110-0109
LRAH36 before corrosion	47.20°	109	(113)	Fe_2_O_3_	COD 96-210-8029
	52.35°	1150	(101)	α Fe	COD 96-110-0109
	54.97°	93	(200)	γ-(FeO(OH)) lepidocrocite	COD 96-901-5157
	77.20°	244	(200)	α Fe	COD 96-110-0109
LRAH36 after corrosion	35.53°	195	(011)	γ-(FeO(OH)) lepidocrocite	COD 96-901-5157
	43.70°	202	(140)	γ-(FeO(OH)) lepidocrocite	COD 96-901-5157
	47.20°	127	(113)	Fe_2_O_3_	COD 96-210-8029
	52.35°	1113	(101)	α Fe	COD 96-110-0109
	54.97°	168	(200)	γ-(FeO(OH)) lepidocrocite	COD 96-901-5157
	77.20°	183	(200)	α Fe	COD 96-110-0109
	87.58°	111	(142)	γ-(FeO(OH)) lepidocrocite	COD 96-901-5157

**Table 6:** Chemical composition of BVDH36 naval steel.

C	Mn	Si	P	S	Al	Cu	Cr	Ni	V	Mo	Fe
[%]	[%]	[%]	[%]	[%]	[%]	[%]	[%]	[%]	[%]	[%]	[%]
0.17	1.19	0.18	0.011	0.006	0.039	0.05	0.02	0.05	0.003	0.008	98.3

**Table 7:** Chemical composition of LRAH36 naval steel.

C	Mn	Si	P	S	Al	Cu	Cr	Ni	V	Mo	Ti	Fe
[%]	[%]	[%]	[%]	[%]	[%]	[%]	[%]	[%]	[%]	[%]	[%]	[%]
0.16	1.20	0.23	0.01	0.011	0.03	0.05	0.03	0.04	0.003	0.006	0.001	98.2

**Table 8:** Physical–chemical characteristics of the natural seawater before corrosion.

Black Sea Water	pH	Conductivity [mS/cm]	Salinity [ppt]
	8.31	22.2	13.4

## Data Availability

Data are contained within the article.
